# Manual of Dietetics

**Published:** 1886-11

**Authors:** 


A Manual of Dietetics. By J. Milner Fothergill, M. D., *Edin.*, *Physician to the City of London Hospital for Diseases of the Chest* (*Victoria Park*); *Hon. M.D. Rush Medical College, Chicago, Ill.; Foreign Associate Fellow of the College of Physicians, Philadelphia, 8vo, extra muslin. pp. 255. New York:* William Wood and Company. *Chicago:* W. T. Keener.

The author of this manual has established a well-deserved popularity among the profession in the United States, which this, his latest, book will in no way diminish. He has been engaged in active practice of his profession for more than thirty years, and, during the last ten, prominently connected with two of the London Hospitals and the Leeds Public Dispensary. His writings have an attractive literary quality of their own, and his opinions never fail to command the respect even of those who occasionally fail to agree with him.

The book is divided into two parts.

The first part treats of the object of food, its forms and their digestion, methods of preparing it, condiments, beverages, stimulants, fluid food, preserved and canned foods, prepared foods, and artificial digestive agents.

This part of the manual is by no means a cook-book, but nevertheless the author has been not only careful to enumerate special dishes, but also to state in a general way how they should be prepared. The discussion of methods of preparation is minute enough for the guidance of any good cook.

The chapters in the second part are short treatises on the proper food in infancy, in adolescence, in adult life, in old age, in acute disease, in convalescence, in gastric affections, in struma, in anæmia, in constipation and diarrhœa, in phthisis, in chronic heart and lung disease, in Bright's disease, in albuminuria, in diabetes, in glycosuria, in gout, in neurosal affections, for chronic invalids, in obesity, in indigestion, in biliousness, and food given otherwise than by the mouth.

The treatment of the topics included in the table of contents is in strict conformity with the latest physiological knowledge, and is minute enough to form a practical working guide in any form of illness. The author's claim that the dietetics is fully as important as the therapeutics of a case will be denied by few who read the book. His statement that "the time indeed is at hand when systematic lectures on food will be a part of medical education," is that of an enthusiast, but in this part of the world it is as yet not realized.

Beef-tea, as ordinarily made, he classes as a simple stimulant, and refuses it any food-value. His remarks in this connection, on pages 49, 50, 51, 66 and 95 are almost facetious, and the following on page 137 is only too true: "Depend upon it, myriads of our fellow-creatures have perished because those around them did not know how to feed them." And this on page 139: "Where beef tea (as ordinarily made) is given alone on the misapprehension that it is a sustaining food, the system has to exist on its own resources, and too often the patient sinks into a grave not dug for him by disease, but by the ignorance of those who constitute the nurses and attendants."

The practical importance of the subject treated in this little book is far greater than that of many a more ponderous volume. It is not only a valuable book, but also one which no doctor, who has the best interests of his patients at heart, can afford not to own and read. It cannot be too highly commended.

We notice the following errors in proof-reading, viz: page 7, line 2, "fu l" for fuel; page 152, line 15, the word "mobility" evidently should read "inability"; page 168, line 6, the word "avoiding" should be omitted, in order to make the statement what the author evidently intended; page 168, line 21, the word "flesh" should be "fresh"; on page 251, the word "ounce" should be substituted for the abbreviation "oz."

